# How Many Words Do We Know? Practical Estimates of Vocabulary Size Dependent on Word Definition, the Degree of Language Input and the Participant’s Age

**DOI:** 10.3389/fpsyg.2016.01116

**Published:** 2016-07-29

**Authors:** Marc Brysbaert, Michaël Stevens, Paweł Mandera, Emmanuel Keuleers

**Affiliations:** Department of Experimental Psychology, Ghent UniversityGhent, Belgium

**Keywords:** word knowledge, vocabulary size, reading

## Abstract

Based on an analysis of the literature and a large scale crowdsourcing experiment, we estimate that an average 20-year-old native speaker of American English knows 42,000 lemmas and 4,200 non-transparent multiword expressions, derived from 11,100 word families. The numbers range from 27,000 lemmas for the lowest 5% to 52,000 for the highest 5%. Between the ages of 20 and 60, the average person learns 6,000 extra lemmas or about one new lemma every 2 days. The knowledge of the words can be as shallow as knowing that the word exists. In addition, people learn tens of thousands of inflected forms and proper nouns (names), which account for the substantially high numbers of ‘words known’ mentioned in other publications.

## Introduction

Researchers dealing with quantitative aspects of language are often asked how many words a typical native speaker knows. The question is raised not only by lay people but also by colleagues from various disciplines related to language processing, development, acquisition, and education. The answer usually starts with a deep sigh, followed by the explanation that the number depends on how a word is defined. As a result, in the literature one finds estimates going from less than 10 thousand to over 200 thousand (see below). In this paper, we try to give practical answers for American English depending on the definition of a word, the language input an individual is exposed to, and the age of the individual.

## Terminology

In this text, we will need a number of terms related to words. For the readers’ ease we summarize them here.

### Word Types vs. Word Tokens

Word types refer to different word forms observed in a corpus; tokens refer to the total number of words in a corpus. If the corpus consists of the sentence “The cat on the roof meowed helplessly: meow meeooow mee-ee-ooow,” then it has nine word types (the, cat, on, roof, meowed, helplessly, meow, meeooow, and mee-ee-ooow) and 10 word tokens (given that the word type “the” was observed twice). Somewhat surprisingly, in some word counts the words “The” and “the” are considered as two different word types because of the capital letter in the first token of “The.” If such practice is followed, the number of word types reported nearly doubles.

### Alphabetical Word Type

This is a word type consisting only of letters. In the example above mee-ee-ooow would be deleted. The cleaning to get to alphabetical word types in addition involves eliminating the distinction between uppercase letters and lowercase letters. So, the words “GREAT” and “great” are the same alphabetical type.

### Lemma

Uninflected word from which all inflected words are derived. In most analyses is limited to alphabetical word types that are seen by the English community as existing words (e.g., they are mentioned in a dictionary or a group of people on the web use them with a consistent meaning). In general, lemmas exclude proper nouns (names of people, places, …). Lemmatization also involves correcting spelling errors and standardizing spelling variants. In the small corpus example we are using, there are six lemmas (the, cat, on, roof, meow, and helplessly).

### Word Family

A group of lemmas that are morphologically related form a word family. The various members are nearly always derivations of a base lemma or compounds made with base lemmas. In our small example corpus the lemmas “the, cat, on, roof, and meow” are all base lemmas of different families, but the lemma “helplessly” can be simplified to “help.”

Below we will see what the various definitions of “words known” mean for vocabulary size estimates. First, we discuss how many words there are to learn (in English).

## In Theory, the Number of Word Types in a Language is Infinite

Kornai (2002) argued that the number of word types in a language is boundless because language users constantly coin new words.^1^ This is linked to the observation that the number of word types increases as a function of the corpus size. All else equal, the number of word types will be smaller in a small corpus than in a large corpus, as new types add up the more words a person (or machine) processes. When the very first words of a corpus are processed, each word is a new type. Very rapidly, however, word types start to repeat (e.g., the word “*the*” occurs in nearly every sentence) and the increase in word types slows down. The more words processed already (i.e., the larger the corpus size), the less likely the next word will be a new type, because most word types have already been encountered.

**FIGURE 1:**
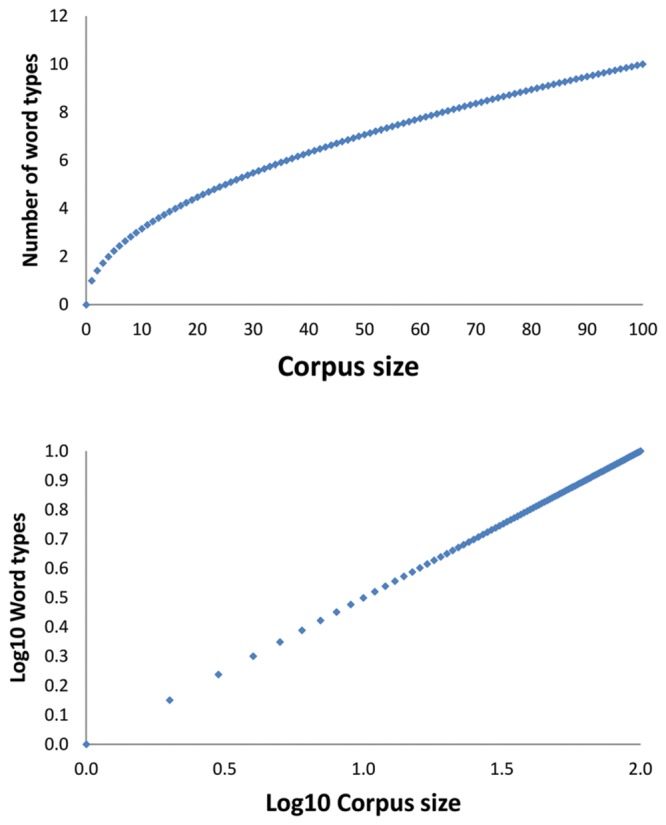
**Figures illustrating Herdan’s or Heap’s law.** The **(Top)** figure shows how the number of word types would increase if the law were a power law with exponent 0.5 (i.e., square root). The **(Bottom)** figure shows the same information when the axes are log_10_ transformed. Then the concave function becomes a linear function, which is easier to work with.

Herdan (1964) and Heaps (1978) argued that the function linking the number of word types to the corpus size has the shape of a power function with an exponent less than 1 (i.e., it will be a concave function). This function is shown in the upper part of **Figure 1** It is known as Herdan’s law or Heap’s law (Herdan described the function first, but Heap’s book had more impact). We will call the function Herdan’s law in the remainder of the text.

Mathematicians prefer to present power functions in coordinates with logarithmically transformed axes, because this changes the concave function into a linear function, which is easier to work with, as shown in the bottom part of **Figure 1** Kornai’s (2002) insight was that if the number of word types is limited, then at a certain point Herdan’s law will break down, because the pool of possible word types has been exhausted. This will be visible in the curve becoming flat from a certain corpus size on.

Kornai (2002) verified Herdan’s law for corpora up to 50 million word tokens and failed to find any flattening of the predicted linear curve, indicating that the pool of possible word types was still far from exhausted. Since Kornai’s (2002) analysis, corpora of vastly greater size have been released and when Brants and Franz (2006) made the first 1.025 trillion word corpus available based on the English internet webpages at that time, they verified that Herdan’s law still applied for a corpus of this size. Brants and Franz (2006) counted 13.6 million word types in their corpus, with no indication of a stop to the growth.

**FIGURE 2:**
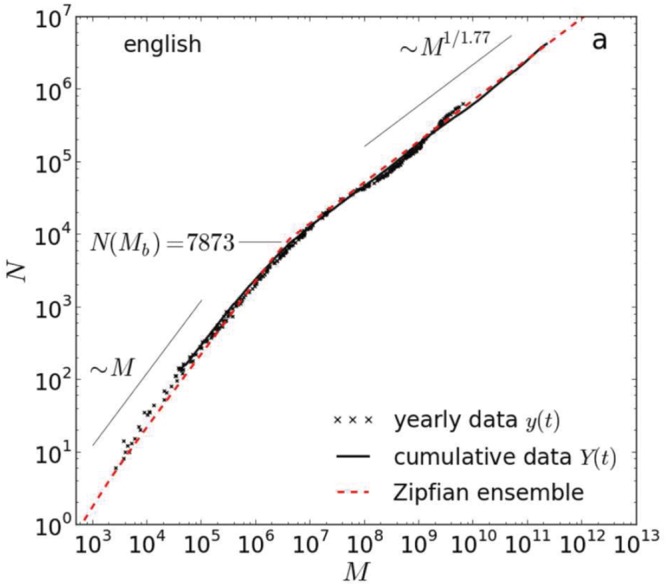
**Herdan’s or Heap’s law applied to the Google books corpus of over 400 billion words.** It shows the number of alphabetical unigrams (N) as a function of the corpus size (M). The individual data points represent the various years in the corpus. The black line is the cumulative corpus over the years. The red line is the growth in N on the basis of the equations derived by Gerlach and Altmann (2013). According to Herdan’s or Heap’s law, the function between M and N should be a straight line (as both scales are logarithmic; base 10). However, the curve shows a discontinuity at *N* = 7,873 (*M* ≈ 3,500,000; see also Petersen et al., 2012). As a result, different equations were proposed for the two parts of the curve. Source: Gerlach and Altmann (2013).

A look at the types in Brants and Franz’s (2006) corpus reveals that a great deal of them consist of alphabetical characters combined with non-letter signs, most of which no native speaker would accept as constituting a word in English (similar to the word “mee-ee-ooow” in the example above). In order to confirm that the growth in types is not the result of these arbitrary combinations of characters, some cleaning is required. One such cleaning is to lowercase the corpus types and limit them to sequences involving the letters a–z only (Gerlach and Altmann, 2013). As indicated above, we call the resulting entries ‘alphabetical word types.’ **Figure 2** shows the increase in the number of alphabetical word types (N) as a function of corpus size (M)^2^ (Gerlach and Altmann, 2013). The data are based on the Google Books corpus of over 400 billion words (Michel et al., 2011).

As can be seen, the curve shows an unexpected change at *N* = 7,873 (*M* ≈ 3,500,000) and then continues with a steady increase up to the full corpus size. Gerlach and Altmann (2013) explained the change at *N* = 7,873 as the point at which the core vocabulary of English is incorporated. As long as this vocabulary is not exhausted, the increase in new word types per 1000 words in the corpus is higher than after this point. A factor contributing to the larger increase of word types in the beginning may be the existence of function words. Function words form a close class of some 300 words, needed for the syntax of the sentence. Most of them are high frequency words (i.e., likely to be encountered early in a corpus).

The equation describing Herdan’s law above the flection point describes the extended vocabulary and is the one we will use for our estimates of the total number of alphabetical types that can potentially be encountered. It is defined as follows:^3^

$$N\;\text{=}\;\text{0}.{\text{0922/41}}^{\text{*}}{\text{3500000}}^{(\text{1}}{{}^{\;}}^{\text{-}}{{}^{\;}}^{\text{1/1}.\text{77})}{}^{\text{*}}M^{\left(\text{1/1}.\text{77}\right)}$$

This equation predicts that there are about 9.6 million alphabetical word types in Brants and Franz’s (2006) corpus of 1.025 trillion words. To arrive at 15 million alphabetical types, a corpus of 2.24 trillion words would be required.

## Toward a Pragmatic Answer 1: How Many Alphabetical Types are People Likely to Have Encountered?

Although it is correct that a language can contain an unlimited number of alphabetical types, this does not address the question how many word types people are likely to know, or, in other words, how large their vocabulary is. For this, we need practical answers to the following two questions: ‘How many alphabetical types are people likely to have encountered in their life?’ and ‘How many of these alphabetical types do we consider as words of a language?’ We will start with the first question.

The number of alphabetical types that people can come across is limited by the speed with which they process language. As we will show, the existence of corpora of hundreds of billions of word tokens should not mislead us into thinking that such exposure is possible in a lifetime. In addition, it is important to consider individual differences: Not everyone consumes language at the same speed. We will distinguish between three theoretical cases: (a) a person who gets all language input from social interactions, (b) a person who gets all language input from television programs, and (c) a person who gets all language input from constant reading. As we will see, this distinction gives rise to major differences in the number of words encountered. In addition, we must consider age: All else equal, older people will have encountered more words.

Mehl et al. (2007) asked a group of male and female students to wear microphones during the day and recorded 30 s of sound of every 13 min (the duration was limited to assure the participants’ privacy). On the basis of the data gathered, Mehl et al. (2007) estimated that their participants spoke about 16,000 word tokens per day, with no significant difference between men and women. Assuming that participants *listened* to the same amount of word tokens, the total input from social interactions would be equal to 16,000 × 2 × 365.25 = 11.688 million word tokens per year. For a 20-year-old, this adds up to about 234 million word tokens (assuming full input from day one)^4^; for a person of 60 years, it grows to slightly more than 700 million word tokens. Applying Gerlach and Altmann (2013) equation, this would predict that a 20-year-old has encountered 84,000 alphabetical word types while a 60-year-old has encountered 157,000 alphabetical word types. In all likelihood, the corresponding vocabularies would be smaller than the number of encountered types. One reason is that word diversity is considerably larger in written materials (on which Gerlach and Altmann’s estimate is based) than in spoken language (Hayes, 1988; Cunningham and Stanovich, 2001; Kuperman and Van Dyke, 2013). Another reason is that one cannot assume all word types to be memorized at their first encounter.

Van Heuven et al. (2014) sampled all subtitles from BBC1, the British public broadcaster’s most popular TV channel. On the basis of this corpus, it can be estimated that the yearly input for someone who constantly watches the channel, is 27.26 million word tokens per year (i.e., more than twice the input from social interactions). This results in a total input of 545 million word tokens for a 20-year-old and 1.64 billion word tokens for a 60-year-old. The numbers are likely to be overestimates, as broadcasts go on for some 20 h per day.

Reading rate is estimated between 220 and 300 word tokens per minute, with large individual differences and differences due to text difficulty and reading purpose (Carver, 1989; Lewandowski et al., 2003). If we take the upper limit and assume that a record reader reads 16 h per day, this gives a total of 300 ^∗^ 60 ^∗^ 16 ^∗^ 365.25 = 105 million word tokens per year. For a 20-year-old this adds up to 2.1 billion word tokens, and a 60-year-old has seen 6.3 billion word tokens. Applying Gerlach and Altmann’s (2013) equation, this would imply that these two people have encountered 292,000 and 543,000 alphabetical word types, respectively.

In summary, based on our assumptions about the amount of tokens encountered in different modalities and on the relationship between word tokens and word types, we can roughly estimate the number of alphabetical word types one has likely encountered: A 20-year-old exposed exclusively to social interaction will have encountered around 81,000 alphabetical types, while a 20-year-old exposed non-stop to text will have encountered around 292,000 different alphabetical types. For a 60-year-old, the corresponding estimates are 157,000 and 543,000 alphabetical types, respectively. As we will see in the next sections, we would not ordinarily consider all these alphabetical types as words of a language.

## Toward a Pragmatic Answer 2: From Alphabetical Types to Lemmas

**Table 1**, which shows an arbitrary extract of the corpus types in the Google Books Corpus (Michel et al., 2011), makes two things clear. First, a large number of alphabetical types encountered in large corpora are proper nouns (names). Unlike common nouns, verbs, adjectives, or adverbs, most proper nouns are understood to mean the same in different languages. In other words, knowledge of proper nouns is independent of knowledge of a particular language. As the notion of a vocabulary ordinarily refers to the body of words used in a particular language, it makes sense to exclude proper nouns from the counts of words known in a language.

**Table 1:** Extract from the word list of Google Books Ngram viewer.

ekam
ekamantam
ekatvam
eke
ekiben
ekistic
ekklesia
ekklesiologische
ekkuklema
ekonomicheskoye
ekonomicznego
ekonomisk
eks
ekstatic
ektexine
ekun
E.K.
EKAW’2000
EKG
EKV

The second thing we see when we look at the list of alphabetical types from a large corpus, is that it contains many alphabetical types reflecting regional differences (e.g., English vs. American English), typographical errors, spelling mistakes, and words from other languages. As we do not consider these to be part of the target language, it is clear that they must be excluded from the word counts as well.

A further reduction is possible by excluding all regular inflections. In general, verbs in English have four forms (‘display, displays, displayed, and displaying’) and nouns have two forms (‘divination and divinations’). Some adjectives have different forms for the positive, the comparative and the superlative (‘gentle,’ ‘gentler,’ ‘gentlest’). The ground form (‘display,’ ‘divination,’ ‘gentle’) is called the lemma.^5^ When a list of vocabulary types in English (excluding names) is lemmatized, the number of lemmas is about 60% of the original list.

A straightforward technique to estimate the number of lemmas in a language is to analyze dictionaries. Goulden et al. (1990), for instance, analyzed Webster’s Third New International Dictionary (1961), chosen because it was the largest non-historical dictionary of English. The preface of the dictionary said it had a vocabulary of over 450,000 words.^6^ When the inflections were excluded, the number decreased to 267,000 lemmas. Of these, 19,000 were names and 40,000 were homographs. The latter are distinct meanings of words already in the list. For instance, words like “bat, bill, can, content, fan, fine, lead, light, long, spring” have more than one entry in the dictionary because they have more than one unrelated meaning. Subtracting the names and the homographs leaves an estimate of 208,000 “distinct” lemma words in the Webster’s Third New International Dictionary (1961). These include multiword expressions, such as phrasal verbs (give in, give up) multiword prepositions (along with) and fixed expressions with a meaning that cannot be derived from the constituting words (kick the bucket).

Another way to estimate the number of lemmas in English in the absence of proper nouns, spelling variants, spelling errors, and unaccepted intrusions from other languages, is to make use of lists designed by people who have a particular interest in compiling a more or less exhaustive list of English words: scrabble players. The Collins Official Scrabble Words (2014) guide contains 267,751 entries, going from ‘aa, aah, aahed, …’ to ‘…, zyzzyvas, zzz, zzzs.’ The Official Tournament and Club Word (OTCW) List from the North American Scrabble Players Association includes only 178,691 entries going from ‘aa, aah, aahed, …’ to ‘…, zyzzyva, zyzzyvas, zzz.’^7^ Since the lists are tailored to scrabble users, they do not contain words longer than 15 letters or shorter than two letters (the minimum number of letters required on the first move in Scrabble). The list includes inflections, however. These can be pruned with an automated lemmatizer (Manning et al., 2008). Then the number of lemmas in the Collins list reduces to some 160,000.

## Toward a Pragmatic Answer 3: From Lemmas to Word Families

When one looks at lists of lemmas, it rapidly becomes clear that they still contain a lot of redundancy, as shown in **Table 2** Words form families based on derivation and compounding. Knowledge of one word from a family helps to understand the meaning of the other members (although it may not be enough to produce the word) and to learn these words.

**Table 2:** Extract from a lemma list showing the existence of word families.

nomad
nomadic
nomadically
nomadism
nomenclatorial
nomenclatural
nomenclature
nominal
nominalist
nominalization
nominally
nominate
nominated
nomination
nominative
nominator
nominee
nomothetic
non
non-absorbent

The power of morphological families has been investigated most extensively in second language education (Goulden et al., 1990), where it was observed that participants were often able to understand the meaning of non-taught members of the family on the basis of those taught. If you know what ‘diazotize’ means, you also know what ‘diazotization, diazotizable, and diazotizability’ stand for, and you have a pretty good idea of what is referred to with the words ‘misdiazotize, undiazotizable, or rediazotization.’ Similarly, if you know the adjective ‘effortless,’ you will understand the adverb ‘effortlessly’ (you can even produce it). You will also quickly discover that the adjective ‘effortless’ consists of the noun ‘effort’ and the suffix ‘-less.’ And if you know the words ‘flower’ and ‘pot,’ you understand the meaning of ‘flowerpot.’

Psycholinguistic research (Schreuder and Baayen, 1997; Bertram et al., 2000) has also pointed to the importance of word family size in word processing. Words that are part of a large family (‘graph, man, work, and fish’) are recognized faster than words from small families (‘wren, vase, tumor, and squid’).

Goulden et al. (1990) analyzed the 208,000 distinct lemma entries of Webster’s Third New International Dictionary (1961) described above. Of these, they defined 54,000 as base words (referring to word families), 64,000 as derived words, and 67,000 as compound words (there were also 22,000 lemmas that could not be classified). The person, who has spent most energy on defining word families, is Nation (e.g., Nation, 2006)^8^. His current list has pruned (and augmented) Goulden et al.’s (1990) list of 54,000 base words to some 28,000 word families, of which 10,000 suffice for most language understanding.

As is the case with all natural categories, the boundary between base words and derived words is not clear (see Bauer and Nation, 1993, for guidelines). Although most of the time the categorization is straightforward, there are a few thousand borderline instances. For instance, what to do with the trio ‘abbey, abbot, and abbess’? They are not morphologically related according to present-day morphological rules, but they are historically related and the similarity in meaning and orthography helps to understand and learn the three of them. A similar question can be asked about ‘move and motion’: Are they part of the same family or not, given their small orthographic and phonological similarity? Authors disagree which of these are base words and which not, leading some scholars to conclude that the transition from lemmas to word families creates more confusion than clarity (Schmitt, 2010). Therefore, it is interesting to do the counts for both definitions.

## How Many Lemmas and Word Families are Known According to the Literature?

When we look at the various attempts to estimate the vocabulary size of an adult (typically an undergraduate student), we clearly see the impact of the various definitions given to “words” (**Table 3**). Nearly all estimates limit words to lemmas, as defined above. In addition, most make some further reduction by using various definitions of “word families.” As a result, the estimates range from less than 10 thousand words known to over 200 thousand words mastered.

**Table 3:** Various estimates of the number of English words known by adults (typically first-year university students), together with the way in which “words” were defined and the task used.

Study	Estimate	Definition of “word”	Task
Hartmann (1946)	215,000	All entries from Webster’s New International Dictionary	Meaning production
Nusbaum et al. (1984)	14,400	Lemmas present both in Miriam-Webster’s Pocket Dictionary and Webster’s Seventh Collegiate Dictionary (list of 19,750 words)	Familiarity rating
Goulden et al. (1990)	17,200	Base words (sic) from Webster’s Third New International Dictionary, excluding proper nouns, derived words, and compounds.	Indicate whether word is known or not
D’Anna et al. (1991)	17,000	Functionally important lemmas (sic) from the Oxford American Dictionary, with the exception of abbreviations, hyphenated words, affixes, contractions, interjections, letters, multiword entries, slang, capitalized entries, foreign words, alternate spellings, and outdated words.	Subjective estimates of knowledge
Anderson and Nagy (1993)	40,000	Distinct lemmas (sic) from a corpus based on school textbooks; excludes proper nouns and a limited number of very transparent derived words and compounds.	Various tests
Zechmeister et al. (1995)	12,000	Same as in D’Anna et al. (1991)	Multiple choice questions related to the meaning of the words
Milton and Treffers-Daller (2013)	9,800	Same as in Goulden et al. (1990)	Provide synonym or explanation for words known

Unsurprisingly, the highest number comes from a study (Hartmann, 1946) in which no pruning of lemmas (or inflections) was done. Hartmann (1946) selected 50 words (one from the same relative position on every fortieth page) from the unabridged *Merriam Webster’s New International Dictionary* (second edition) and administered the words to 106 undergraduate students. The participants were asked to supply the meanings of the words without time limit. Given that the dictionary contained 400 thousand words and the students on average were able to give definitions for 26.9 of the 50 words, Hartmann concluded that they had a vocabulary of 215,000 words. As we saw above, this estimate includes proper nouns, inflected forms, derived words and compounds.

Goulden et al. (1990) also started from (a later edition of) Webster’s International Dictionary but reduced the number of “interesting words” to the 54,000 base words discussed above. They selected 250 of these words and presented lists of 50 to a total of 20 university graduates, who were native speakers of English and over the age of 22. Students simply had to indicate whether they knew the word. The estimated number of basic words known ranged from 13,200 to 20,700 with an average of 17,200.

Milton and Treffers-Daller (2013) used the same words as Goulden et al. (1990) but asked participants to give a synonym or explanation for each word they knew. Participants were mainly first year university students. With this procedure and population Milton and Treffers-Daller (2013) obtained an estimate of 9,800 word families known.

## How Many Lemmas and Word Families are Known? a New Study

To supplement the existing estimates, we ran a new study on a much larger scale, both in terms of words tested and in terms of people tested.

### Stimulus List

As the authors before us, we rapidly came to the conclusion that not all words in the Webster dictionary and the Collins scrabble lists are of interest, because they include many names of plants, insects, and chemical substances, which come close to proper nouns for non-specialists. In addition, creating a stimulus list on the basis of copyright protected sources creates a problem for free distribution of the materials, which is needed if one wants science to be cumulative.

To tackle the above issues, we decided to build a new list of lemmas ‘worthwhile to be used in psycholinguistic experiments,’ based on word frequency lists (in particular the SUBTLEX lists from Brysbaert et al., 2012 and Van Heuven et al., 2014) and free lists of words for spell checkers. The main criterion for inclusion was the likely interest of non-specialists in knowing the word. Compared to the Collins scrabble list, the list does not include the lemmas ‘aa, aah, aal, allii, aargh, aarrgh, aarrghh, aarti, aasvogel, ab, aba, abac, abacterial, abactinal, abactor, …, zymolysis, zymome, zymometer, zymosan, zymosimeter, zymosis, zymotechnic, zymotechnical, zymotechnics, zymotic, zymotically, zymotics, zythum, zyzzyva, and zzz.’ It does, however, include the words ‘a, aardvark, aardwolf, abaca, aback, abacus, …, zymogen, zymology, and zymurgy.’ All in all, the list contains 61,800 entries and is attached as Supplementary Materials to the present article. Although the list was compiled for research purposes, we are fairly confident it contains the vast majority of reasonably known English words (in American English spelling)^9^, but we agree that it would be possible to add about the same number of more specialist words.

To see where we would get and to go for the maximum difference between the lemma list and the word family list, we decided to interpret the family size of our 61,800 lemma list maximally. That is, all words that could reasonably be derived from a base word were considered to be part of the base word’s family. Compound words were split in their constituting families, unless the meaning of the compound could not be derived from the constituents (as in ‘honeymoon, huggermugger, and jerkwater’). This resulted in 18,269 word families (see the Supplementary Materials). With less strict criteria, the list could easily be enhanced to 20,000 families or even 25,000.^10^ On the other hand, while reading the list of families, one is constantly tempted to prune even further (so that a list of 18,000 may be achievable as well). The basic finding, however, is that English words boil down to a list of building blocks not much larger than 20,000 words, when names and acronyms (which are often names as well) are excluded.^11^ The rather small number of word families is testimony to the tremendous productivity of language.

### Participants and the Vocabulary Test Used

To see how many of our list of 61,800 lemmas are known, we presented them to participants with a test similar to the one used by Goulden et al. (1990). Because of the massive internet availability nowadays, it was possible to present the entire list of words to a much larger and heterogeneous group of people (Keuleers et al., 2015).

The test we ran^12^ follows the design described in Keuleers et al. (2015). First, participants were asked a few questions, the most important of which for the present discussion were about their age, whether English was their native language, and whether they took higher education. After answering the questions, the participants were shown a random list of 67 words and 33 non-words and asked to indicate which words they knew. The non-words were generated with the Wuggy algorithm (Keuleers and Brysbaert, 2010). Participants were warned about the non-words and they were told they would be punished for yes-responses to these letter strings. To correct the scores for guessing, the proportion of yes-responses to the non-words was subtracted from the proportion of yes-responses to the words. So, someone who selected 50/67 words and said yes to 1/33 non-words, obtained a score of 0.746 - 0.033 = 0.713, indicating that they knew 71.3% of the words. Across participants, data were collected for the full list of 61,800 lemmas and over 100,000 non-words.

Participants taking part in the vocabulary test consented to their data being used for scientific analyses at the word level. At no point they were asked to identify themselves, so that data gathering and data use were not linked to individuals. Participation was voluntary, did not put strain on the participants, and could be stopped at any time. This procedure is in line with the General Ethical Protocol followed at the Faculty of Psychology and Educational Sciences of Ghent University.

All in all, we tested 221,268 individuals who returned 265,346 sessions. The number of sessions is higher than the number of participants because quite a few participants took the vocabulary test more than once (each session had a different sample of words and non-words). In order not to give undue weight to individuals (some participants took the test more than 100 times), we limited the analyses to the first three sessions if participants completed multiple sessions.

## Results

**FIGURE 3:**
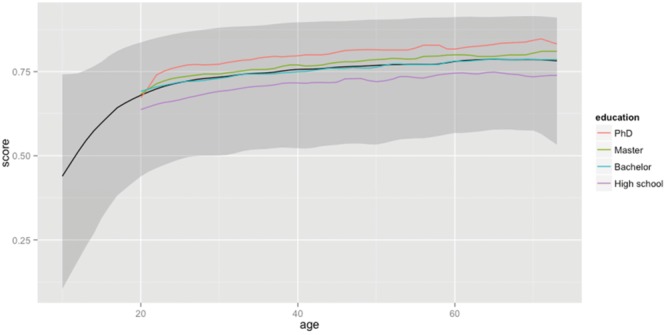
**Percentage of lemmas known as a function of age and educational level.** The solid black line shows the median percentage known as a function of age. It shows a steady increase up to the age of 70 (for the people who took part in the study). The gray zone indicates the range of percentages between percentile 5 and percentile 95. The impact of education level is shown in the lines representing the medians of the various groups.

**Figure 3** shows the percentage of lemmas known to native speakers of American English as a function of age and education level (see Keuleers et al., 2015, for similar findings in Dutch). As can be seen, the knowledge of words increases with age and education. As a matter of fact, the effect of age very strongly resembles the power function that would be expected on the basis of Herdan’s law (**Figure 1**): Because older people have come across more words than younger people, they also know more words. This suggests that people rarely forget words, even when they don’t know the (exact) meaning any more. Indeed, a test in which persons are asked to spot a word among non-words is thought to be a good estimate of premorbid intelligence in elderly people and patients with dementia (Baddeley et al., 1993; Vanderploeg and Schinka, 2004; but also see Cuetos et al., 2015 for evidence that dementia patients are deficient at spotting words among non-words, in particular words that are low in frequency, low in imageability, and acquired later in life).

The median score of 20-year-olds is 68.0% or 42,000 lemmas; that of 60-year-olds 78.0% or 48,200 lemmas. This corresponds to a difference of 6,200 lemmas in 40 years’ time (or about one new lemma every 2 days). The difference between education levels is also substantial and likely illustrates the impact of reading and studying on vocabulary knowledge. Indeed most of the difference between the education levels seems to originate during the years of study.

**FIGURE 4:**
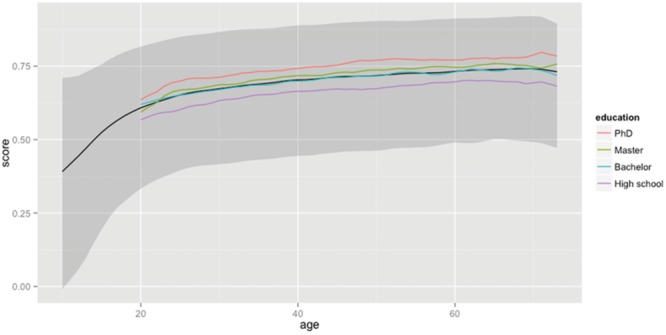
**Percentage of base words (word families) known as a function of age and educational level.** The solid black line shows the median percentage known as a function of age. It shows a steady increase up to the age of 70 (for the people who took part in the study). The gray zone indicates the range of percentages between percentile 5 and percentile 95. The impact of education level is shown in the lines representing the medians of the various groups.

**Figure 4** shows the same information for the base words (the word families).^13^ An interesting observation here is that the overall levels of word knowledge are lower. A 20-year-old knows 60.8% of the base words (for a total of 11,100 base words), and a 60-year-old knows 73.2% (or 13,400 base words). The lower percentage of base word knowledge is to be expected, because well-known lemmas tend to come from large word families. So, the 325 lemmas including ‘man’ (craftsmanship, repairman, congressman, …) are better known than the four lemmas including ‘foramen’ (foramen, foraminiferous, foraminifera, and foraminifer). The former add much more weight to the tally of lemmas known (325 vs. 4) than to the tally of base word known (1 vs. 1). As a result, quite a lot of lemmas can be known based on the mastery of a limited number of prolific base words.

## The Estimates

The findings so far are summarized in **Table 4** and translated into reasonable estimates of words known. They show that the estimates depend on (a) the definition of word known, (b) the age of person, and (c) the amount of language input sought by the person. For the number of alphabetical types encountered, the low end is defined as a person who only gets input from social interactions; the high end is a person who constantly reads at a pace of 300 words per minute. For the number of lemmas and base words known the low end is defined as percentile 5 of the sample we tested, the median as percentile 50, and the high end as percentile 95.

**Table 4:** Estimates of the words known by 20-year-olds and 60-year-olds at the low end and the high end.

Person	Number of alphabetical types encountered	Number of lemmas known (max = 61,800)	Number of base words known (max = 18,300)
**20-year-old**			
Low end	84,000	27,100	6,100
Median		42,000	11,100
High end	292,000	51,700	14,900
**60-year-old**			
Low end	157,000	35,100	9,000
Median		48,200	13,400
High end	543,000	56,400	16,700

The number of lemmas known arguably is what most people spontaneously associate with the answer to the question ‘how many words are known.’ The number of base words (word families) mastered indicates that these lemmas come from a considerably smaller stock of building blocks that are used in productive ways. Notice that the average number of words known by a 22-year-old (17,200), as estimated by Goulden et al. (1990), lies in-between our estimated number of lemmas known (42,000) and our estimated number of base words known (11,100), in line with the observation that Goulden et al. (1990) used less invasive criteria to define their base words.

Multiplying the number of lemmas by 1.7 gives a rough estimate of the total number of word types people understand in American English when inflections are included.^14^ The difference between alphabetical types encountered and the number of inflected forms known gives an estimate of names and unknown words people see in their lives. As Nation (2006) argued, it is easier to understand how a 20-year-old could have learned 11,100 base words (which amounts to 1.7 new words per day if learning commences at the age of 2) than to understand how they could have acquired 1.7^∗^42,000 = 71,400 new word forms (inflections included), which amounts to nearly 11 new words per day (see Nagy and Anderson, 1984, for such an estimate).

The estimates of **Table 4** are for receptive vocabulary (understand a word when it is presented to you). Productive word knowledge (being able to use the word yourself) is more limited and estimated to be less than half receptive knowledge. The difference between receptive and productive word knowledge increases as the words become less frequent/familiar (Laufer and Goldstein, 2004; Schmitt, 2008; Shin et al., 2011).

## Limitations

It is unavoidable that the estimates from **Table 4** are approximations, dependent on the choices made. All we can do, is be transparent about the ways in which we derived the figures and to make our lists publicly available, so that other researchers can adapt them if they feel a need to do so.

A first limitation is the list of 61,800 lemmas we used. Although we are reasonably sure the list contains the vast majority of words people are likely to know, there are ample opportunities to increase the list. As indicated above, the Collins scrabble list could be used to more than double the number of entries. We are fairly confident, however, that such an increase will not change much in the words known by the participants (see also Goulden et al., 1990). The words we are most likely to have missed are regionally used common words and recently introduced words. On the other hand, a recent study by Segbers and Schroeder (2016) in German illustrates the importance of the word list started from. These authors worked with a list of 117,000 lemmas and on the basis of a procedure similar to Goulden et al. (1990) concluded that native German adults know 73,000 lemmas of which 33,000 are monomorphemic (which comes closest to our definition of base lemmas). A comparison of both lists by a German–English researcher would be informative to see why the estimates are larger in German than in English. German has more single-word compounds than English (which partly explains the higher number of lemmas known in German than in English), but this should not affect the number of monomorphemic lemmas known.

A second limitation is that our list does not include meaningful multiword expressions. Expressions such as ‘have to, give in, washing machine, salad spinner, kick the bucket, at all, …’ were excluded from our lemma list. Such sequences are particularly important when the meaning of the expression is not clear from the individual words. Martinez and Schmitt (2012) reported 505 such non-transparent expressions among the 5,000 most frequent words in English, suggesting that the estimates of **Table 4** should be increased by 10% to include multiword expressions that cannot be derived from the constituent words.

A third limitation is that our definition of words does not take into account the fact that some words have multiple senses and sometimes even meanings. Words like ‘mind, miss, sack, second’ have several meanings that are unrelated to each other. Goulden et al. (1990) estimated that 15 percent of the entries in Webster’s Third were homographs (word with multiple meanings), of which two thirds did not differ substantially from the previous entry (i.e., the difference was a difference in sense rather than meaning). So, a reasonable estimate would be that 5% of the words are ambiguous in their meaning and our measure has no way to assess whether the participants were familiar with the various meanings. Even worse, our assessment says nearly nothing about how well the participants know the various words. They were only asked to select the words they knew. Estimates of vocabulary size are smaller if more demanding tests are used, as can be seen in **Table 3** [e.g., compare the estimate of Zechmeister et al. (1995) to that of D’Anna et al. (1991) and the estimate of Milton and Treffers-Daller (2013) to that of Goulden et al. (1990)]. Indeed, understanding the meaning of words is not a binary, all-or-nothing phenomenon but a continuum involving multiple aspects (Christ, 2011). As such, our estimates should be considered as upper estimates of word knowledge, going for width rather than depth. The estimates also deal with receptive word knowledge (understanding words other people use). As indicated above, productive knowledge is thought to be roughly half of receptive word knowledge.

Finally, our list excludes names (and acronyms). An interesting question is how many names people are likely to know. In principle, these could run in hundreds of thousands (certainly for older people reading a lot, as shown in **Table 4**). On the basis of our experiences, however, we believe that the number is more likely to be in the tens of thousands or even thousands (depending on the person). For instance, when we probed a large segment of the Dutch-speaking population about their knowledge of fiction authors with a test similar to the vocabulary test described above, we saw that few people know more than 500 author names (out of a total of 15,000 collected from a local library).^15^ Hidalgo and colleagues set up a website about world famous people^16^. Thus far, the number includes some 11,000 names. It would be interesting to examine how many of these are effectively known by various people. The smaller estimates agree with the observation of Roberts et al. (2009) that the number of social contacts people have is limited to some 150, although of course people know many more names (of humans and places) than the individuals they have personal relationships with.

## Conclusion

Based on an analysis of the literature and a largescale crowdsourcing experiment, we estimate that an average 20-year-old student (the typical participant in psychology experiments) knows 42,000 lemmas and 4,200 multiword expressions, derived from 11,100 word families. This knowledge can be as shallow as knowing that the word exists. Because people learn new words throughout their lives, the numbers are higher for 60-year-olds. The numbers also depend on whether or not the person reads and watches media with verbal content.

## Author Contributions

All authors listed, have made substantial, direct and intellectual contribution to the work, and approved it for publication.

## Conflict of Interest Statement

The authors declare that the research was conducted in the absence of any commercial or financial relationships that could be construed as a potential conflict of interest.

## Footnotes

1 At the same time, the number of word types in a language is combinatorially limited by phonotactic constraints and practical limits on word length. As a conservative example, a language with 10 consonants and five vowels would have 550 possible CV and CVC syllables. Combining those syllables would result in more than 50 trillion possible word types up to five syllables long. Our use of the term boundless should be considered in this light.

2 The Google Books Ngram database has a lower limit of 41 occurrences before a word is included in the database. This roughly corresponds to a frequency of 1 per 10 billion words and decreases the number of unigrams drastically, given that about half of the unigrams observed in corpus have a frequency of 1 (Baayen, 2001). A similar threshold was used by Brants and Franz (2006).

3 Notice that 1/1.77 = 0.565, so that the increase in N as a function of M above *N* = 7,873 is slightly higher than the square root of M.

4 Notice that this number is an upper limit, given that people do not have full input from day one and given that they are unlikely to produce meaningful new words when they speak themselves. Readers are free to calculate alternative estimates if they do not feel comfortable with the definitions we use.

5 Not all verb forms are inflections, as some are used frequently as adjective (appalled) or noun (cleansing). Also noun plurals can have a different meaning and, therefore, lemma status (aliens, glasses, and minutes). For more information, see Brysbaert et al. (2012).

6 This number is higher still in later editions of the dictionary and it is not difficult to find claims of even more than twice this number. According to a company that monitors English websites, Global Language Monitor, the English language has nearly 1.050 million words, with 14.7 new words created each day (all having a minimum of 25,000 citations from various geographical regions; (http://www.languagemonitor.com/number-of-words/number-of-words-in-the-english-language-1008879/, August, 13, 2015). Needless to say, the vast majority of the entries are names and transparent derivations, compounds and multiword expressions.

7 The Collins list also includes slang words, such as devo, lolz, obvs, ridic, shizzle, and inflections of derivations, such as abjectnesses and abnormalisms.

8 http://www.victoria.ac.nz/lals/about/staff/paul-nation

9 If readers know of interesting words not included in the list, please send them to MB.

10 Nation (2006) reduced the word list of the British National Corpus (100 million words) to 82,000 lemmas and 28,000 word families, keeping words like ‘nominee, non-entity, norovirus, northbound, and northern’ as separate word families rather than assigning them to the families ‘nominate, entity, virus, and north.’

11 There are only five words of 16 letters in the list of basic words (caryophyllaceous, chryselephantine, prestidigitation, tintinnabulation, and verticillastrate). All others are in the scrabble list.

12 http://vocabulary.ugent.be/

13 For the calculation, knowledge of word families consisting of bound morphemes (zymo-, -blast, …) was estimated on the basis of the full word with the highest recognition rate (zymology, blastocyst, etc).

14 If 60% of the English word forms are lemmas and 40% inflected forms, the total number of words can be derived from a lemma list by multiplying the number of lemmas by 1/0.60 = 1.7.

15 In hindsight, we could have expected this number. Given that few people read more than one book per week, the total input for a 20-year-old is only 1040 books (if they started reading from day one). In addition, several of these books will come from the same authors.

16 http://pantheon.media.mit.edu/
